# Lay Counselor Perspectives of Providing a Child-Focused Mental Health Intervention for Children: Task-Shifting in the Education and Health Sectors in Kenya

**DOI:** 10.3389/fpsyt.2019.00860

**Published:** 2019-12-17

**Authors:** Shannon Dorsey, Rosemary D. Meza, Prerna Martin, Christine L. Gray, Noah S. Triplett, Caroline Soi, Grace S. Woodard, Leah Lucid, Cyrilla Amanya, Augustine Wasonga, Kathryn Whetten

**Affiliations:** ^1^Department of Psychology, University of Washington, Seattle, WA, United States; ^2^Center for Health Policy and Inequalities Research, Duke Global Health Institute, Duke University, Durham, NC, United States; ^3^Department of Global Health, University of Washington, Seattle, WA, United States; ^4^Ace Africa- Kenya, Bungoma, Kenya; ^5^Terry Sanford Institute of Public Policy, Duke University, Durham, NC, United States

**Keywords:** global mental health, implementation science, task-shifting, acceptability, appropriateness, feasibility, evidence-based treatment

## Abstract

The global mental health treatment gap has increasingly been addressed using task-shifting; however, very little research has focused on lay counselors’ perspectives on the acceptability, feasibility, and appropriateness of mental health interventions in specific government-supported sectors that might scale up and sustain mental health care for children and adolescents. In western Kenya, these sectors include Education and Health. Data come from a large hybrid effectiveness-implementation study examining implementation practices and policies in either or both sectors that support successful implementation of a child-focused intervention, Trauma-focused Cognitive Behavioral Therapy (TF-CBT), for children and adolescents who had experienced parental death. We examined lay counselors’ self-report of acceptability, feasibility, and appropriateness of TF-CBT. Lay counselors were teachers (n = 30) from the Education sector and Community Health Volunteers (CHVs; n = 30) from the Health sector, who were part of Sequence 1 of a large stepped-wedge, cluster randomized trial. Lay counselor self-report surveys included reflective and formative measurement of acceptability, feasibility, and appropriateness administered after lay counselors in both sectors had experience delivering the locally-adapted, group-based TF-CBT intervention. Descriptive statistics (means, standard deviations) were used to understand counselors’ perspectives stratified by sector. Both teachers and CHVs endorsed high acceptability, feasibility, and appropriateness of TF-CBT, with lay counselors’ responses on items from the formative measures providing some insight into specific aspects of acceptability, feasibility, and appropriateness that may be important to consider when planning for implementation support. These early findings suggest that both sectors may hold promise for task-shifting of mental health care for children and adolescents but also underline the importance of considering the multiple facets of these three implementation outcomes as well as lay counselor context (Education vs. Health).

## Introduction

Mental health disorders have increasingly become recognized as a prominent cause of global morbidity, with a significant portion of the world’s population—450 million people—affected (1). In a recent reassessment of global burden of disease, mental illness accounted for 32.4% of years lost to disability (YLDs) and ranked on par with cardiovascular and circulatory diseases, each responsible for 13% of disability-adjusted-life-years (2). Children, adolescents, and young adults 5 to 24 years of age bear a high burden of mental illness. It is the leading cause of YLDs for this age group (3) and 75% of mental disorders commence during this period of life (4). Poverty is associated with increased susceptibility to mental illness, so consequently low- and middle-income countries (LMICs) are disproportionately affected (5). While services are improving, substantial gaps remain. The treatment gap is as high as 78% for adults (6) and even greater for children, with a median of only 0.16 of children in LMICs with mental health illness receiving any treatment (7). Services target mainly adults and severe mental illness, resulting in children being overwhelmingly neglected. Only 12% of outpatient and 6% of all other types of mental health facilities serve children (8). Most LMICs allocate 1-2% of the government’s health budget to mental health services, which translates to less than $0.25 per year per capita (9). The funding gaps are correspondingly accompanied by shortage in human resources, as demonstrated by the relatively low mental health staff ratio of 1 per 100,000 population compared to more than 50 staff per 100,000 population in high-income countries (HICs; 10).

Given limited funding for mental health, and particularly children’s mental health, sustainable services likely need to leverage existing systems and require few new resources (11). Task-shifting, a process in which non-specialists with little to no prior training or experience provide treatment under supervision (12), is recommended as a strategy to address mental health workforce gaps. A growing body of evidence documents the effectiveness of task-shifted mental health services across a range of LMICs and diverse cultural contexts (e.g. 13–17). A 2013 Cochrane review concluded that lay counselors have the potential to improve outcomes for depression or posttraumatic stress disorder (PTSD), but more research is needed, especially in regards to child treatment by lay counselors (18). In another systematic review focused specifically on the acceptability and feasibility of task-shifting among service users and healthcare practitioners (19), insufficient resources was identified as a primary barrier. Task-shifting strategies that leverage existing government-supported systems and require few new resources are necessary in resource-constrained settings. For children and adolescents, these systems include Education and Health, with delivery by individuals [e.g., teachers; community health volunteers (CHVs)] already part of these systems. However, little is known about these individuals’ perspectives on delivering mental health treatment in their systems. Understanding lay counselors perspectives and experiences delivering mental health care in their systems is important so that we can understand potential for scale up and sustainment as well as any needed supports for scale up and sustainment.

The overall goal of this paper is to examine intervention acceptability, feasibility, and appropriateness among the first lay counselors who delivered a trauma-focused mental health intervention for children and adolescents in two distinct sectors in western Kenya. For the purpose of this paper, we use Proctor and colleagues’ (20) definitions of the constructs of interest: Acceptability is conceptualized as the perception among stakeholders that an intervention is agreeable or satisfactory. Lack of intervention acceptability is a well-established barrier to implementation (21). Feasibility is the extent to which an intervention can be successfully carried out within a given context. Appropriateness is defined as the perceived relevance or compatibility of an intervention to a given setting and problem. While these constructs are related, each is conceptually distinct and may have different implications for implementation. For example, an intervention could have high appropriateness but low feasibility in a low-resource setting.

The global mental health literature includes only a few empirical studies of lay counselor perspectives on acceptability, feasibility, and/or appropriateness of delivering mental health interventions. At the time of the review by Padmanathan and De Silva (19), they identified only one study that examined lay counselor perspectives on intervention delivery, conducted by Jordans and colleagues. The authors found that 5–47% (varied by country) of counselors (N = 694) in a multilayered psychosocial intervention for children in areas of conflict in Burundi, Sudan, Sri Lanka, and Indonesia experienced distress when delivering the intervention (22). Lay counselors at different levels of the intervention received different training and supervision support; the least distress was endorsed by the group that received the most training and support. Since Padmanathan and De Silva’s 2013 review, research in this area has increased, but there are still gaps in our knowledge. For example, in a 2014 qualitative study of Zambian lay counselors’ experience providing Trauma-focused Cognitive Behavioral Therapy (TF-CBT; 23) (N = 19), counselors drawn from a wide variety of settings described experiences consistent with high perceived acceptability (e.g., liking TF-CBT; seeing positive impacts on children and families); however, counselors also reported challenges, including treatment duration and poor attendance (24, 25). Post-training practice and supervision groups were noted as helpful and motivating. In a study focused specifically on the feasibility, acceptability, and effectiveness of a training for teachers on providing psychoeducation and facilitating access to mental health care in Haiti (*N* = 22; 26), both qualitative and quantitative data indicated the training was acceptable and feasible, though participants recommended extending the duration and number of training sessions.

Of the existing studies of lay counselors, few have been able to separately examine perspectives of lay counselors nested within specific government sectors and even fewer have focused on child and adolescent interventions. An additional limitation has been when and how constructs have been measured. Studies often have investigated these constructs only during what Aarons and colleagues (27) define as the exploration and preparation phases of implementation, and have not included an evaluation once counselors have actual experience delivering the intervention/s. Due to the limited availability of quantitative measures, most studies have used a qualitative approach to understanding counselor perspectives (e.g., 24). While qualitative approaches provide rich data about counselor experiences, they require intensive resources and time that some mental health implementation efforts may not have and may limit comparison across studies. Only recently have quantitative scales been developed to assess these constructs (28, 29), with their use potentially allowing for expanded research in this area.

The goal of the present study is to assess counselor-perceived acceptability, feasibility, and appropriateness of a trauma-focused intervention for children and adolescents in the context of a task-shifting, implementation science-focused study in western Kenya. We specifically examine these constructs with the first lay counselors from two different sectors, Education and Health, who delivered TF-CBT (23) for children who experienced the death of one or both parents and have related mental health symptoms (30, 31). While these first lay counselors represent a sub-group of our final sample (60 of 240), given study design and randomization procedures, they likely are representative of counselors in the full sample. In addition, our sub-sample is larger than most other studies examining these constructs in lay counselors. Given limited global mental health research focused on implementation science outcomes and on children and adolescents, findings from this sub-group of 60 lay counselors are valuable.

We evaluate lay counselor perspectives after the counselors had sufficient experience implementing the intervention. Our measurement approach distinguishes two types of assessment that facilitate a deeper understanding of the constructs of acceptability, feasibility, and appropriateness. For acceptability and feasibility, we use combined reflective measurement—where covariation in items reflects variation in some underlying construct—and formative measurement—where variation in items is thought to cause a change in a construct (see 32 for a comprehensive review of reflective and formative measurement). For appropriateness, we use formative measurement at both the provider- and organizational-level. A blended reflective-formative approach allows for understanding of both counselors’ overall sense of these constructs, and specific experiences that provide insight into their perceptions. We explore differences between counselor perspectives across the two sectors, given differences in regard to counselor workload, resources, and role/embeddedness in their organizations.

## Method

### Building and Sustaining Interventions for Children (BASIC) Overview

Data for this study come from a large NIMH-funded hybrid effectiveness-implementation study of TF-CBT delivered through both the Education (via teachers) and Health (via CHVs) sectors in Western Kenya: Building and Sustaining Interventions for Children (BASIC; NIMH-funded R01MH112633) conducted as a collaboration between Duke University, the University of Washington, and Ace Africa, in Bungoma, Kenya (33). This study builds on a pilot of TF-CBT in East Africa (31) and a large randomized controlled trial (RCT) in Kenya and Tanzania (30) that demonstrate the effectiveness of lay counselor-delivered TF-CBT in Kenya. The goal of BASIC is to learn what makes an enabling context for mental health delivery in both Education and Health, by identifying implementation practices and policies (IPPs) that support successful implementation in the distinct sectors. The study is a stepped wedge cluster randomized trial (SW-CRT) that includes seven sequences (i.e., sites that initiate the intervention in the same time period). Per best practice for SW-CRT reporting (34) we use SW-CRT CONSORT language here, to describe the design. In Kanduyi Constituency, there are 137 primary schools, each with an active health extension program in the surrounding community. We randomly selected and ordered 40 primary schools and their surrounding 40 communities (i.e., 40 “village clusters”) into seven sequences, which initiate delivery of the TF-CBT intervention at different time points over the 5-year study. The first sequence, which is the focus of this study, included 10 village clusters (10 schools and 10 communities). Sequences 2–7 each have five village clusters. The first sequence involved more village clusters (10 vs. 5) so that findings from TF-CBT implementation in these schools and communities could inform implementation in subsequent sites.

The Ministries of Education and Health are involved in BASIC, provide stakeholder input and permission to schools, teachers, and CHVs to participate, and receive updates from schools, communities, and Ace Africa so that they can monitor progress. Village leaders are also involved, with CHVs sensitizing chiefs and village elders about the intervention so that they can encourage children and families to participate and reduce any perceived stigma. BASIC has three aims. Aim 1 involves identifying locally feasible and effective IPPs from the first 10 schools and 10 communities that implemented TF-CBT. These findings, from the first 25% of the sample, were used to develop implementation support for subsequent sites (i.e., the remaining 30 schools and 30 communities). Aim 2 of BASIC examines IPPs that predict adoption, fidelity, and sustainment in the full sample of 40 village clusters (40 schools and 40 communities). Aim 3 examines child clinical outcomes and implementation costs. The full BASIC sample will include 240 counselors (120 teachers, 120 CHVs) from 40 primary schools and 40 communities.

### Procedures

Data for this study come from counselor self-report surveys from the first 10 village clusters randomly assigned to Sequence 1 in BASIC. Teacher and CHV counselors were trained in TF-CBT in January 2018 (see *Lay Counselor Training* below). They delivered two sequential TF-CBT groups (one focused on girls, one focused on boys) during Term 1 and Term 2 of the academic year. Counselors participated in self-report surveys that assessed their demographic characteristics and experience prior to training. They also completed self-report surveys assessing their beliefs and perceptions about TF-CBT immediately post-training and after implementing the two TF-CBT groups (i.e., post-implementation). This study uses the latter, post-implementation counselor self-report measures of acceptability, feasibility and appropriateness as we believed their ratings would be more informed after gaining experience delivering TF-CBT. This data was collected between July and August 2018. The teacher counselor surveys were administered in English, whereas CHV counselor surveys were administered in Kiswahili to accommodate language preferences. All procedures were approved by the Institutional Review Boards (IRBs) at Duke University and the Kenya Medical Research Institute.

### Participants

Participants were 60 lay counselors (30 teachers; 30 CHVs) from the first 10 schools and 10 communities who delivered TF-CBT as part of BASIC. Three counselors were selected for each site, as counselors worked in a group of three to deliver the intervention; two counselors led the child group and one counselor led the guardian group. Ace Africa worked with Head Teachers in each of the selected schools to identify teachers who would be appropriate for delivering TF-CBT. Ace Africa also worked with Community Health Extension Workers to identify CHVs from their health facility who worked as part of health extension in the communities surrounding the schools. Leaders were asked to nominate three individuals who were good with children, may have had some counseling experience (but not required), have time to deliver the program each week, and who have no immediate plans for leaving their school/area (e.g., ideally in the same school or village for two years). Demographic and background characteristics are presented in **Table 1**. Teachers and CHVs received an incentive of 500 Kenyan Shillings, approximately 5 USD, for each interview.

**Table 1:** Characteristics of Teacher Counselors and Community Health Volunteers (CHVs) Counselors from 10 clusters in the trial.

Characteristic	Teachers (*N* = 30)		CHVs (*N* = 30)	
	*N*	%	*N*	%
Gender				
Female	21	70%	21	70%
Male	9	30%	9	70%
**Highest Level of Education**				
None	0	0%	0	0%
Primary education	0	0%	7	23%
Secondary education	2	7%	22	73%
Certificate	17	57%	1	3%
Diploma Certificate	3	10%	0	0%
University Degree	0	0%	0	0%
Master’s Degree	8	27%	0	0%
**Received any prior training in psychosocial counseling**				
Yes	16	53%	18	60%
No	14	47%	12	40%
	**Mean**	**SD**	**Mean**	**SD**
**Age in Years**	42.8	7.7	44.5	9.5
				
**Years as a Teacher or CHV**	17.8	9.3	7.4	4.4
				
**Years in the school or community where you currently work or volunteer**	9.1	8.3	7.7	4.7

CHV, Community Health Volunteer; SD, Standard Deviation.

### Intervention

TF-CBT is an evidence-based treatment protocol which treats psychosocial sequelae from child trauma exposure, with a specific application for maladaptive grief (23). There is substantial evidence of its effectiveness from RCTs in high-income countries (HICs; 35) with evidence of effectiveness from large RCTs in Zambia (15) and Kenya (30) and two small RCTs in the Democratic Republic of Congo (36, 37). In our previous collaborative work, we modified TF-CBT for delivery in Eastern Africa, with results from an open trial in Tanzania suggesting that the intervention held promise, given positive clinical outcomes at the end of treatment and maintained gains at a 1-year follow up (31). The modified TF-CBT, called *Pamoja Tunaweza* (Together We Can) involved 12 weeks of weekly group sessions and 3–4 individual sessions. Group-based delivery was chosen for multiple reasons: a) all youth were receiving treatment for the same primary traumatic event (parental death); b) the group format maximized reach and efficiency (i.e., more children served); and c) early qualitative work suggested that a group-based intervention was perceived as more culturally appropriate, more supportive, and less stigmatizing. Following the open trial, we conducted a large RCT in eastern Tanzania and western Kenya (*N* = 640), testing *Pamoja Tunaweza* compared to usual care services at end of treatment and a 1-year follow-up (30). The intervention was delivered by six lay counselors in each country, who were trained and supervised by the experienced lay counselors from the open trial in Tanzania, with support from the first author, following the Apprenticeship Model of training (38).

In BASIC, *Pamoja Tunaweza* included eight group-based sessions (reduced from 12) and two to three individual sessions (39). This abbreviated intervention was piloted with children assigned to usual care, after their 1-year follow up. All groups were delivered at the local schools, which are community owned. Children and guardian groups met concurrently with conjoint activities included in the final four sessions. Each session included practicing new skills at home. Initial group sessions (1–2) focused on psychoeducation about the *Pamoja Tunaweza* program, grief and loss, and on building coping skills. Guardians learned the same information both to reinforce lessons at home and to apply lessons in their own lives. Guardians also learned non-physical skills for managing behavior problems. The individual sessions, provided in-between group sessions 3–5, focused on imaginal exposure/trauma narrative (TN) work. Each child met individually with a counselor from the child group to talk about the parent(s)’ death and surrounding events (e.g., getting sick, funeral). The counselor then met individually with the guardian to prepare them to provide emotional support when the child shared their TN. Group sessions 4–5 involved developing individualized plans for situational exposure (i.e., facing trauma reminders [pictures of the parent/s who died]), supporting TN work, processing trauma-related cognitions, and a group session in which each child individually and privately shared their TN with their guardian (with counselors providing support). The final group sessions (6–8) focused on grief-specific activities (e.g., converting the relationship from interaction to memory).

### Lay Counselor Training

Teachers and CHVs participated in separate, 6-day, *Pamoja Tunaweza/*TF-CBT trainings. Training and supervision were provided by experienced, local Kenyan *Pamoja Tunaweza/*TF-CBT lay counselors from the large RCT, hereafter referred to as the local trainers who worked for Ace Africa. Training involved didactic instruction, manual review, trainer modeling of group sessions, time for questions, and trainee practice of group sessions in small groups, with peer and trainer feedback. For approximately 2–3 weeks following the training, teachers and CHVs practiced delivering *Pamoja Tunaweza/*TF-CBT groups with their co-counselors, with support and supervision from the local trainers.

### Implementation, Supervision, and Fidelity Monitoring

Following practice, teachers and CHVs began delivering *Pamoja Tunaweza/*TF-CBT to children. In brief, children enrolled were those attending the local school in the village cluster, who had experienced the death of one or both parents (and other types of traumatic events), and had either posttraumatic stress symptoms or prolonged grief, determined by mental health assessment tools with locally defined cutoffs from prior research in the region (see 33). All counselors received ongoing, weekly supervision throughout the implementation period. Supervision involved in-person meetings and/or phone calls and SMS interactions *via* Whats App between the local trainers and counselors. Supervision typically included role play and practice by counselors of upcoming *Pamoja Tunaweza/*TF-CBT sessions, with supervisor feedback. When supervision was not in person, supervisors often listened to a role play *via* phone or reviewed an uploaded audio or video role play. Supervisors regularly observed group sessions to monitor fidelity, with a goal of observing 50% of sessions, and completed fidelity ratings for both adherence and competence, two important dimensions of fidelity (40). Counselors completed brief reports of fidelity and individual child/guardian treatment response (e.g., engagement in session or home practice; emotional state in groups) for each session. Supervisors’ group observation and review of counselor reports informed supervision.

### Measures

#### Measurement Approach

**Figure 1:**
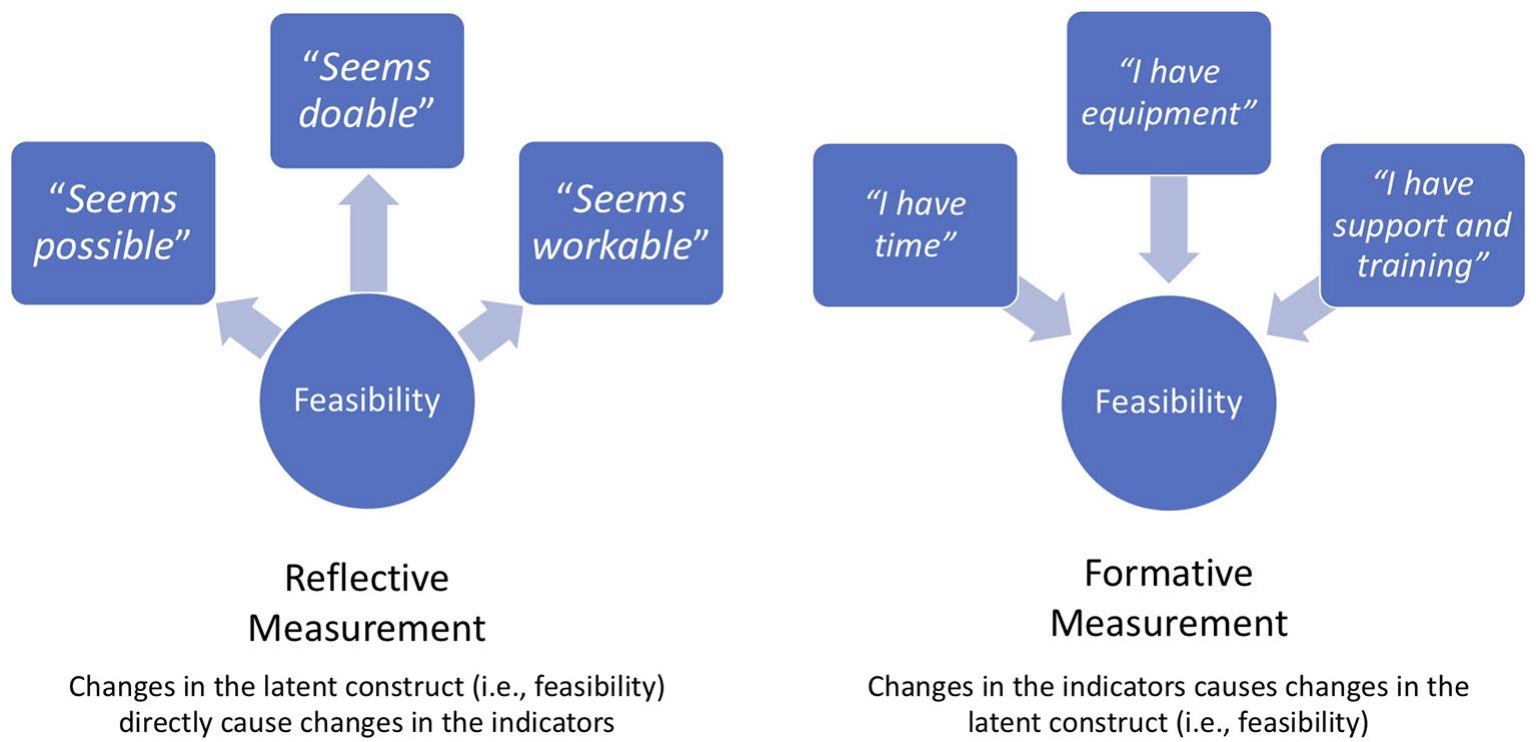
Distinction between reflective and formative measurement models.

We employ a blended reflective-formative approach to measurement of our constructs of interest. Reflective measures follow a principal factor latent variable model—covariation among measures is caused by, and therefore reflects, variation in some underlying latent factor or construct (32). Formative measures follow a composite latent variable model, in which changes in the items are hypothesized to cause changes in the underlying construct. Unlike reflective measures, formative measures do not assume all items reflect a single underlying construct or factor. Rather, they assume that each item has an impact on a construct (32). Because of the nature of reflective measures, the mean score is thought to reflect the underlying constructs. However, reflective scales do not offer any indication of *why* they were rated as they were; thus, formative measures may complement reflective measures to provide depth or context regarding the construct being measured. We use Weiner and colleagues’ (29) reflective measures to assess intervention acceptability and feasibility. We include items from the counselor-level version of the Johns Hopkins University (JHU) Implementation Measure (28), developed specifically for assessing implementation constructs in LMICs, as formative measures, providing more depth and contextual understanding. We also created some new items to assess aspects of feasibility and appropriateness that were not covered by the JHU measures. The reflective measures ask similar questions about the intervention’s acceptability and feasibility (e.g., “*Pamoja Tunaweza/*TF-CBT met my approval” and “*Pamoja Tunaweza/*TF-CBT was appealing”) as a means of assessing the respondent’s perception of the underlying construct of acceptability or feasibility. The formative measures ask about specific elements of the intervention and surrounding context that contribute to its acceptability, feasibility and appropriateness. As such, lay counselors’ responses to individual items in the formative measure provide insight into why they might have rated the reflective measure in the way that they did. Please see **Figure 1** for a graphic representation of reflective and formative measurement.

#### Selection and Adaptation of Reflective Measures

Weiner and colleagues’ (29) newly developed measures assessing intervention acceptability, feasibility and appropriateness were designed using participants from HICs who were mental health professionals experienced in mental health service delivery. Given differences in our context, each of the three measures along with their construct definitions (20) were reviewed in English with supervisors trained in TF-CBT at Ace Africa. Items were translated into Kiswahili by a bilingual supervisor. Another Ace Africa team member who had not seen the original English items back-translated the measure. For the Appropriateness measure, there were more perceived challenges for fit with a task-shifting context. Given the differences in provider types (professionals in HICs with experience delivering different types of mental health treatments vs. lay counselors in LMICs new to mental health treatment delivery of any treatment type), the Weiner (29) Intervention Appropriateness measure did not seem to capture the broader need to assess the appropriateness of the individual’s role or setting for delivering the treatment. Therefore, only the Weiner (2017) Intervention Acceptability and Feasibility measures were used as reflective measures. Inter-item correlations (IICs) for each measure were examined; a minimum threshold of 0.20 was used to retain items (41).

##### Counselor Demographics and Background Characteristics

The questionnaire was administered prior to *Pamoja Tunaweza*/TF-CBT training and assessed counselor gender, age, education level, and previous training and experience providing psychosocial counseling.

##### Acceptability

Two measures were used to assess acceptability. The four-item reflective Acceptability of Intervention measure (29) was used to assess lay counselor perspectives of intervention acceptability. Weiner and colleagues’ (29) reported acceptable internal consistency (α = 0.85) and test-retest reliability (r = 0.80). Internal consistency for the 4-item scale in our sample was 0.68 for teachers and 0.69 for CHVs. The IICs supported retaining all four items, with all above 0.20 at post-implementation.

The JHU Implementation Science scale for Acceptability was used as a formative measure of acceptability, including only the 5 items that mapped directly onto Proctor’s definition of acceptability and did not conceptually overlap with items on the Acceptability of Intervention measure. A validity study of the consumer-level version (vs. counselor/provider-level) of the JHU Implementation Science scale found acceptable test-retest reliability (rho: 0.70) and support for criterion validity (28). While not used as a reflective measure in our study, the counselor-level measure had acceptable internal consistency reliabilities. Analysis of data from our sample suggest convergent validity between the Acceptability of Intervention measure and the individual 5-items on the JHU Implementation Science scale (range for teachers: *r*: 0.36 - 0.59; range for CHVs: *r*: 0.41 - 0.58). One of the five items from the formative scale assessed the acceptability of supervision (e.g., “I was satisfied with the supervision I received when I provided *Pamoja Tunaweza/TF-CBT*”), given the importance of this practice for high-fidelity TF-CBT delivery. Other example items included: 1) the extent to which the lay counselors felt positively about providing *Pamoja Tunaweza*/TF-CBT (e.g., “I felt good about providing *Pamoja Tunaweza*/TF-CBT”) and 2) the extent to which they understood *Pamoja Tunaweza*/TF-CBT (e.g., “I felt that the components of *Pamoja Tunaweza*/TF-CBT made sense to me”) (see **Table 2** for all items). All acceptability items from both the reflective and formative measures were assessed on a 5-point scale ranging from 1 (strongly disagree) to 5 (strongly agree).

**Table 2:** Reflective and Formative Measures of Acceptability among Teachers and CHVs at Post-Implementation.

	Teachers (*N* = 30)			CHVs (*N* = 30)		
Reflective Measure^1^	Mean	SD	Range	Mean	SD	Range
Acceptability of Intervention^2^	4.67	0.34	(4,5)	4.77	0.31	(4,5)
**Items from Formative Measures** **^1^**						
I liked providing PT/TF-CBT	4.63	0.49	(4,5)	4.87	0.35	(4,5)
						
I felt good about providing PT/TF-CBT	4.73	0.45	(4,5)	4.83	0.38	(4,5)
						
I continue to enjoy learning PT/TF-CBT	4.69	0.47	(4,5)	4.90	0.31	(4,5)
						
I felt that the components of PT/TF-CBT made sense to me	4.73	0.52	(3,5)	4.77	0.43	(4,5)
						
I was satisfied with the supervision I received when I provided PT/TF-CBT	4.50	0.82	(1,5)	4.70	0.47	(4,5)

CHV, Community Health Volunteer; SD, Standard Deviation; PT/TF-CBT, Pamoja Tunaweza/Trauma-focused Cognitive Behavioral Therapy.

^1^Items measured on a five-point scale ranging from 1 (strongly disagree) to 5 (strongly agree).

^2^Mean of four items; range reflects minimum and maximum across all four items.

##### Feasibility

Two measures were used to assess feasibility. The 4-item reflective Feasibility of Intervention measure (29) was used to assess lay counselor perspectives of intervention feasibility. Weiner and colleagues’ (29) reported acceptable internal consistency (α = 0.89) and test-retest reliability (r = 0.88). Internal consistency for the 4-item scale in our sample ranged from 0.70 to 0.92 depending on rater (teacher, CHV). The IICs from teacher surveys and all but two correlations from CHV surveys were above 0.20 at post-implementation. Potential translation issues may explain why two IICs were lower on the CHV surveys. Given the smaller sample size of 30 CHVs in Sequence 1, we decided to retain all four items at this time and re-run these analyses with the full sample after the data collection is complete to determine if any questions should be dropped from the CHV survey.

The JHU Implementation Science scale for Feasibility was used as a formative measure of feasibility. A validity study of the consumer-level version (vs. counselor/provider-level) of the JHU Implementation Science scale found acceptable test-retest reliability (rho: 0.76) and support for criterion validity (28). While not used as a reflective measure in our study, the counselor-level measure had acceptable internal consistency reliabilities. Analysis of data from our sample suggest convergent validity between the Feasibility of Intervention measure and the individual items on the JHU Implementation Science scale for CHVs (range for CHVs: *r*: 0.25 - 0.59). Among teacher respondents, the Feasibility of Intervention measure and 7 of the 10 items on the JHU Implementation Science scale suggest convergent validity (range for teachers: *r*: 0.21 - 0.60). Three items had no association (*r* = 0.051 – 0.16) and are noted in **Table 3**. We used 12 of the original 20 items across four domains related to feasibility of intervention delivery: 1) provider skills (e.g., “I believe I am sufficiently skilled at providing *Pamoja* Tunaweza/TF-CBT to orphans”), 2) time (e.g., “I have enough time for all the activities that go into providing *Pamoja Tunaweza*/TF-CBT”), 3) resources (e.g., “I have the right equipment [e.g., pens/pencils/chalk, paper, exercise books, slip charts, mark pens] to regularly provide *Pamoja Tunaweza*/TF-CBT”), and 4) personnel/supervision (e.g., “I am able to reach my *Pamoja Tunaweza*/TF-CBT supervisor when needed”) (see **Table 3** for all items).

**Table 3:** Reflective and Formative Measures of Feasibility among Teachers and CHVs at Post-Implementation.

Reflective Measure^1^	Teachers (*N* = 30)			CHVs (*N* = 30)		
	Mean	SD	Range	Mean	SD	Range
Feasibility of Intervention^2^	4.35	0.60	(2,5)	4.67	0.40	(2,5)
Items from Formative Measures^1^						
I believe I am sufficiently skilled at providing PT/TF-CBT to orphans	4.60	0.72	(2,5)	4.80	0.41	(4,5)
I have enough time for all the activities that go into providing PT/TF-CBT	4.07	0.58	(2,5)	4.57	0.50	(4,5)
I have enough time to spend in supervision activities (e.g., attending supervision, practicing) related to PT/TF-CBT	4.00	0.74	(2,5)	4.60	0.50	(4,5)
I have enough time to travel to and from PT/TF-CBT groups	NA	NA	NA	4.57	0.50	(4,5)
I am provided with necessary transportation to regularly provide PT/TF-CBT	NA	NA	NA	4.17	0.91	(2,5)
I have the right equipment (e.g., pens/pencils/chalk, paper, exercise books, flip charts, etc.)	3.70^3^	1.15	(1,5)	3.43	1.19	(1,5)
I have the resources (e.g., phone, talk time) to reach my clients and/or PT/TF-CBT supervisor in between sessions when needed	4.03^3^	0.89	(2,5)	3.83	0.91	(2,5)
I have sufficient access to a private space to meet with orphans and guardians receiving PT/TF-CBT	4.43	0.68	(2,5)	4.63	0.49	(4,5)
I have access to a space for individual visits with orphans and guardians	4.23	0.63	(2,5)	4.60	0.50	(4,5)
I am able to reach my PT/TF-CBT supervisor when needed	4.67	0.55	(3,5)	4.57	0.68	(2,5)
I have sufficient access to continued PT/TF-CBT intervention support and training	4.33^3^	0.84	(1,5)	4.60	0.50	(4,5)
I have access to the emotional support I may need to handle any stress related to delivering PT/TF-CBT (e.g., hearing stories about their parent death)	4.37	0.56	(3,5)	4.50	0.57	(3,5)
PT/TF-CBT is too complex to do in this school/community	1.90	0.71	(1,4)	1.50	0.73	(1,4)
Average hours per week on PT/TF-CBT (e.g., preparing, delivering, reports, visits)	3.42	1.35	(1,6)	5.13	3.03	(1,12)

CHV, Community Health Volunteer; SD, Standard Deviation; PT/TF-CBT, Pamoja Tunaweza/Trauma-focused Cognitive Behavioral Therapy; NA, Not Applicable.

^1^Items measured on a five-point scale ranging from 1 (strongly disagree) to 5 (strongly agree).

^2^Mean of four items; range reflects minimum and maximum across all four items.

^3^These items did not correlate highly with the reflective, Feasibility of Intervention Measure.

We selected items in each of the 4 domains to best capture the factors most likely to impact feasibility of delivering a group-based trauma intervention for children in these two contexts. For example, we omitted items about certain resources or forms of payment that were not relevant to our settings. Minor changes were also made to the wording of items to fit the local context. All feasibility items from both the reflective Weiner and formative JHU measures were assessed on a 5-point scale ranging from 1 (strongly disagree) to 5 (strongly agree).

In addition to these two measures, we also included two items assessing important aspects of feasibility. The first assessed intervention complexity specifically, given that intervention complexity is inversely related to feasibility (“*Pamoja Tunaweza*/TF-CBT is too complex to do in my school”). This item was measured on a five-point scale ranging from 1 (strongly disagree) to 5 (strongly agree). The second inquired about the estimated hours per week that respondents felt *Pamoja Tunaweza*/TF-CBT would require, given the importance of this information for understanding added workload and feasibility for providers in the two contexts (“On average, how many hours per week do you spend on *Pamoja Tunaweza*/TF-CBT [e.g., preparing for sessions, delivering sessions, and supervision]?”).

##### Appropriateness

Only a formative measure was used to assess appropriateness. We adapted six items from the JHU implementation measures that aligned with Proctor and colleagues’ (20) definition of appropriateness. Minor changes were made to the wording of items to fit the local context (e.g., changing general terms like “job description” to be specific to the counselors’ context: “as part of my role as a teacher [or CHV]”). The validity study of the consumer-level JHU measure found acceptable test-retest reliability (rho: 0.79) and support for criterion validity (28). While not used as a reflective measure in our study, the counselor-level measure had acceptable internal consistency reliabilities. Two additional items were developed to measure appropriateness domain content for which JHU items did not exist. Given challenges in creating new items, we used Hujig’s Theoretical Domains Framework when possible to guide item creation (42). In the resulting 8-item measure, four items assessed the perceived fit of delivering *Pamoja Tunaweza*/TF-CBT with one’s role (e.g., “I believe that teachers/CHVs should be providing *Pamoja Tunaweza*/TF-CBT”). The additional four items assessed the perceived fit of delivering *Pamoja Tunaweza*/TF-CBT in the respective delivery setting (e.g., “*Pamoja Tunaweza*/TF-CBT fits with my [community (CHV) or school (teacher)]’s approach to helping orphaned children”) (see **Table 4** for all items). All appropriateness items were assessed on a 5-point scale ranging from 1 (not at all appropriate) to 5 (extremely appropriate).

**Table 4:** Formative Measure of Provider and Organizational Appropriateness reported by Teachers and CHVs at Post-Implementation.

	Teachers (*N* = 30)			CHVs (*N* = 30)		
Provider Level Appropriateness^1^	Mean	SD	Range	Mean	SD	Range
I believe that I should be providing PT/TF-CBT	4.23	0.68	(3,5)	4.83	0.38	(4,5)
I believe that teachers/CHVs should be providing PT/TF-CBT	4.43	0.68	(3,5)	4.90	0.40	(3,5)
From my perspective, providing PT/TF-CBT is something I feel I should be doing as part of my job	4.47	0.73	(3,5)	4.83	0.46	(3,5)
From my perspective, attending supervision for PT/TF-CBT is something I feel I should be doing as part of my role as a teacher/volunteer activities as a CHV	4.43	0.68	(3,5)	4.67	0.71	(3,5)
**Organizational Level Appropriateness** **^1^**						
I believe that the school/community should be responsible for providing psycho-social education (including psycho-social counseling, psycho-social support or mental health treatment) for orphaned children	4.33	0.80	(3,5)	4.73	0.64	(3,5)
PT/TF-CBT fits with our school/community’s approach to helping orphaned children	4.13	0.78	(3,5)	4.93	0.25	(4,5)
Providing PT/TF-CBT fits with the goals of my school/community	4.13	0.78	(3,5)	4.93	0.25	(4,5)
Providing PT/TF-CBT will be useful for my school/community	4.57	0.63	(3,5)	5.00	0.00	(5,5)

CHV, Community Health Volunteer; SD, Standard Deviation; PT/TF-CBT, Pamoja Tunaweza/Trauma-focused Cognitive Behavioral Therapy.

^1^Items measured on a five-point scale ranging from 1(not at all) to 5(extremely).

### Analytic Approach

Given the sample size of 60 counselors, we followed best practices for small samples and did not conduct null hypothesis significance testing (43, 44). Rather, we used descriptive statistics to understand provider-level perceptions of key implementation measures following delivery of the *Pamoja Tunaweza*/TF-CBT intervention.

For the reflective measures (Intervention Measures for Acceptability and Feasibility; 29), we first averaged each counselor’s scores of the individual items to create the composite score. Individual counselors’ average scores are presented in box plots to provide visualization of overall variation within each site, by sector. We then calculated means (M) and standard deviations (SD) across all teachers and CHVs, respectively. We report means and SDs, as well as the range of scores using the minimum and maximum reported values of any of the items within the scale. Higher scores represent more favorable responses.

For the formative measures (JHU Implementation scales for acceptability and feasibility, additional created items for provider-level and organizational-level appropriateness), we calculated the means and standard deviations of the individual items. We also report the range for each item. As with the reflective measures, higher scores represent more favorable responses. The same method (mean, SD, range) was used for the additional item assessing complexity. Because complexity represents the inverse of feasibility, lower scores reflect more favorable responses for this item. For average number of hours per week, we calculated the mean, SD, and range.

All data were stratified by sector (teachers in Education; CHVs in health) to reflect perceptions of the intervention situated within the two different contexts. Given our study goal of describing counselor perspectives on TF-CBT in both sectors, we did not conduct statistical comparisons across sectors. Consistent with best practices when sample size is small, we did not conduct statistical comparisons across time points (post-training to post-implementation). All analyses were conducted using Stata 14 (45).

## Results

### Counselor Demographics

Our sample included the 30 teachers and 30 CHVs who delivered *Pamoja Tunaweza*/TF-CBT in the first 10 village clusters (Step 1) of the BASIC trial (**Table 1**). Teacher counselors were mostly female (70%), held a certificate (57%) or a Master’s degree (27%), and were on average 42.8 (SD = 7.7) years old. CHV counselors were mostly female (70%), had completed primary (23%) or secondary (73%) education, and were on average 44.5 (SD = 9.5) years old. More than half (53% of teachers and 60% of CHVs) reported receiving some prior psychosocial training although no counselors had prior experience with TF-CBT or other evidence-based interventions for child and adolescent mental health problems.

### Acceptability

**Figure 2:**
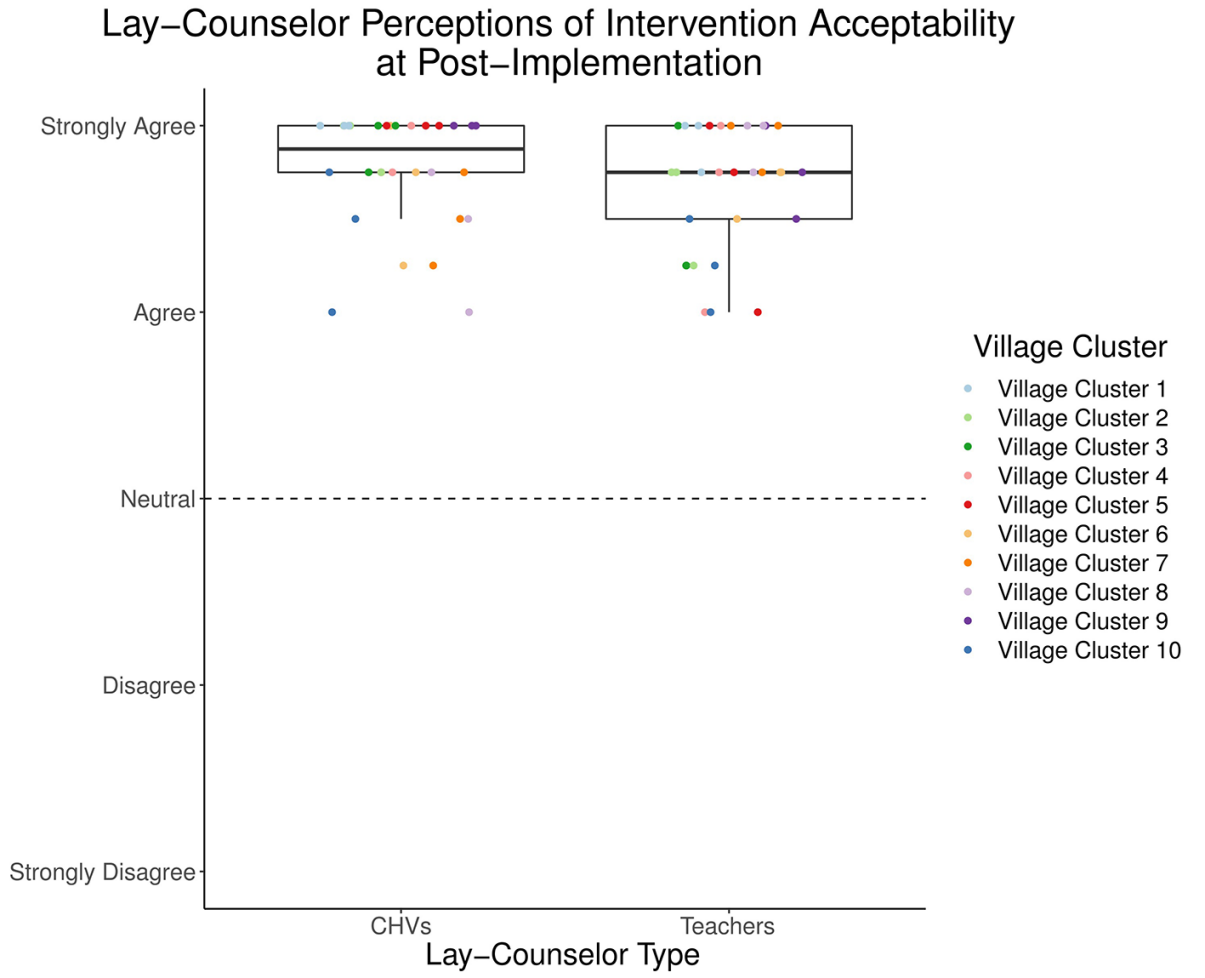
Lay counselor perceptions of intervention acceptability at post-implementation.

The reflective measure (29) indicated high perceived acceptability of the TF-CBT intervention after implementing two groups (post-implementation) for lay counselors in both the Education and Health sectors (**Figure 2** and **Table 2**). Mean acceptability was 4.67 (*SD* = 0.34) for teachers and 4.77 (*SD* = 0.31) for CHVs. Descriptive statistics for all items from the formative measure (28) are included in **Table 2**, separated by counselor type. Examining responses on specific items to understand aspects of acceptability, teachers and CHVs endorsed high agreement with “I felt good about providing *Pamoja Tunaweza/*TF-CBT” (*M* = 4.73, *SD* = 0.45; *M* = 4.83, *SD* = 0.38; respectively). All responses were in the 4 (“agree”) to 5 (“strongly agree”) range. Lay counselors also endorsed high agreement with “I felt that the components of *Pamoja Tunaweza/*TF-CBT made sense to me” (*M* = 4.73, *SD* = 0.52; *M* = 4.77, *SD* = 0.43, respectively), with responses in the 3 (“neither agree nor disagree”) to 5 range for teachers and the 4–5 range for CHVs. Most teachers (*M* = 4.5, *SD* = 0.82; Range 1–5) and all CHVs (*M* = 4.7, *SD* = 0.47; Range 4–5) endorsed high agreement with “I was satisfied with the supervision I received when I provided *Pamoja Tunaweza/*TF-CBT.”

### Feasibility

**Figure 3:**
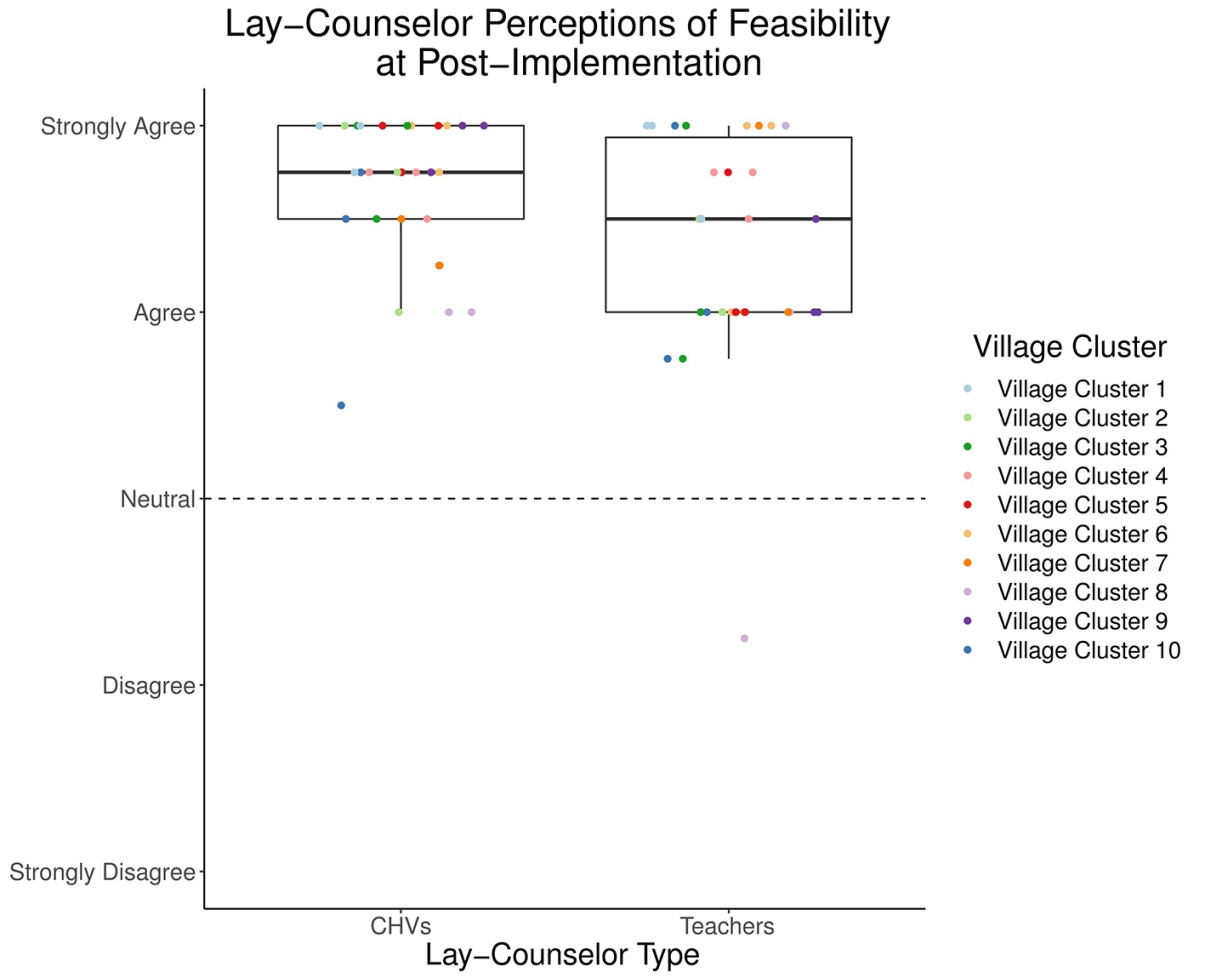
Lay counselor perceptions of intervention feasibility at post-implementation.

The reflective measure (29) indicated high levels of perceived feasibility at post-implementation among teachers (*M* = 4.35, *SD* = 0.60) and CHVs (*M* = 4.67, *SD* = 0.40) (**Figure 3** and **Table 3**). Descriptive statistics for all items from the formative measure are included in **Table 3**, separated by counselor type. Examining responses on specific items from the formative measure (28) to understand aspects of feasibility, both teachers and CHVs expressed high agreement with *“*I believe I am sufficiently skilled at providing *Pamoja Tunaweza/TF-CBT*” at post-implementation (*M* = 4.60, *SD* = 0.72; *M* = 4.80, *SD* = 0.41, respectively), with all but two responses in the 4 (“agree”) to 5 (“strongly agree”) range. Although both teachers and CHVs also expressed high agreement with *“*I have enough time for all activities that go into providing *Pamoja Tunaweza*/TF-CBT” (*M* = 4.07, *SD* = 0.58; *M* = 4.57, *SD* = 0.50, respectively), responses from teachers were more varied (2–5 range; 2 corresponding to “disagree”) than those for CHVs (4–5 range). Among the most highly endorsed statements for both teachers and CHVs was *“*I am able to reach my *Pamoja Tunaweza*/TF-CBT supervisor when needed” (*M* = 4.67, *SD* = 0.55; *M* = 4.57, *SD* = 0.68, respectively). Looking across all items on the formative measure, all but two items at post-implementation had average responses in the 4 (“agree”) to 5 (“strongly agree”) range. Two items within the resources domain were rated the lowest and had the greatest variability across respondents. Both teachers and CHVs rated this item the lowest at post-implementation: “I have the right equipment (e.g., pens/pencils/chalk, paper, exercise books, slip [flip] charts, mark pens) to regularly provide *Pamoja Tunaweza/TF-CBT*” (*M* = 3.70, *SD* = 1.15; *M* = 3.43, *SD* = 1.19, respectively). Another relatively lower-rated resource item, by both counselor types, was: “I have the resources (e.g., phone, talk time) to reach my clients and/or *Pamoja Tunaweza*/TF-CBT supervisor in between sessions when needed” (*M* = 4.03, *SD* = 0.89; *M* = 3.83, *SD* = 0.91 respectively).

One item assessed intervention complexity, “*Pamoja Tunaweza*/TF-CBT is too complex to use in this school/community,” with both teachers and CHVs endorsing disagreement (*M* = 1.90, *SD* = 0.71; *M* = 1.50, *SD* = 0.73, respectively) (**Table 3**). All but six counselors endorsed 1 (“strongly disagree”) or 2 (“disagree”). Another question assessed “On average, how many hours per week do you spend on *Pamoja Tunaweza*/TF-CBT (e.g., preparing for sessions, delivering sessions, and supervision)” (**Table 3**). CHVs reported more weekly hours spent on *Pamoja Tunaweza*/TF-CBT (*M* = 5.13, *SD* = 3.03, Range = 1–12) than teachers (*M* = 3.42, *SD* = 1.35, Range = 1–6) at post-implementation.

### Appropriateness

Descriptive statistics for all items from the formative measure are included in **Table 4**. Examining provider-referenced items at post-implementation, teachers and CHVs endorsed high agreement with “I believe that teachers/CHVs should be providing *Pamoja Tunaweza/TF-CBT*” (*M* = 4.43, *SD* = 0.68; *M* = 4.90, *SD* = 0.40, respectively), with responses in the 3–5 range for both groups. At the mean level, most teachers and CHVs also endorsed high agreement with “From my perspective, providing Pamoja Tunaweza/TF-CBT is something I feel I should be doing as part of my [job duties]” (*M* = 4.47, *SD* = 0.73; *M* = 4.83, *SD* = 0.46, respectively). Nearly all CHVs “strongly agreed” whereas 60% of teachers “strongly agreed” and more teachers endorsed a lower rating of “moderately.” Examining organization-referenced items at post-implementation, teachers and CHVs endorsed high agreement with “Providing *Pamoja Tunaweza*/TF-CBT will be useful for my school/community.” All teacher responses were in the 3 (“moderately”) to 5 (“extremely”) range (*M* = 4.57, *SD* = 0.63); all CHVs responded to this item with a 5 (“extremely”). Teachers and CHVs also endorsed high agreement with “Providing *Pamoja Tunaweza*/TF-CBT fits with the goals of my school/community,” although there was more variability in teachers’ responses (*M* = 4.13, *SD* = 0.78; *M* = 4.93, *SD* = 0.25, respectively).

## Discussion

### Overall

Lay counselors in both Education and Health endorsed high acceptability, feasibility, and appropriateness of TF-CBT after implementing *Pamoja Tunaweza*/TF-CBT. Our results provide preliminary evidence that lay counselors in both sectors perceived TF-CBT to be a satisfactory intervention (acceptability), doable in their respective settings (feasibility), and that delivering TF-CBT is compatible with their role and sector’s approach to helping children (appropriateness). Our findings suggest that using task-shifting to deliver mental health services through Education and community health extension in western Kenya is a promising approach for potential scale up and sustainment.

The formative measures provide some context as to why lay counselors in both sectors rated TF-CBT favorably and also may suggest some differences across lay counselors from the two sectors. The high ratings for acceptability among lay counselors in both sectors are notable given demographic differences in these groups and differences in their primary roles. For instance, teachers generally reported more advanced education levels than CHVs, yet both endorsed favorable ratings with regard to TF-CBT making sense to them and feeling sufficiently skilled in TF-CBT delivery. Our findings mirror those from other studies, in which lay counselors with varied educational backgrounds have competently delivered multi-component mental health interventions that include some complex elements (e.g., cognitive processing; 46). At least among this sample of the first implementing schools and communities in BASIC, our findings are aligned with Murray and colleagues (24) who found high TF-CBT acceptability among Zambian lay counselors. In our study, there appears to be greater variability in satisfaction with the supervision received among teachers compared to CHVs.Other research notes the importance of supervision, particularly for task-shifting (22). It will be important for our research team, and other groups undertaking implementation efforts involving task-shifting, to quickly identify any challenges with supervision as well as identify any factors that may be associated with lower satisfaction with supervision. Teachers’ responses on other formative items from the feasibility scale may provide some insight into factors that can be investigated with the larger sample (e.g., high endorsement for ability to reach the supervisor when needed, lower endorsement for having the time to engage in supervision).

Overall, counselors seem to perceive TF-CBT to be highly feasible, but there was more variation in feasibility, compared to acceptability and appropriateness, and there appears to be some variability across the two counselor types. Not surprisingly given the resource-constrained context, both teachers and CHVs seem to rate the availability of resources needed both to deliver the intervention (e.g., paper, chalk) and communicate with clients and supervisors (e.g., phone credit) lower than other aspects of feasibility. These findings reflect Padmanathan and De Silva’s (2013) identification of resources as a primary barrier to task-shifting mental health care, and underline the importance of planned implementation coaching for subsequent schools and communities in BASIC (33). While we did not engage in formal statistical testing of differences, there appear to be some differences in how feasibility was perceived across the two sectors. Teachers’ perceptions of feasibility, although still high, had greater variability than did CHV perceptions (**Figure 3**). Teachers were delivering TF-CBT and finding time to participate in supervision on top of their full-time positions as teachers and were often constrained by schools’ schedules. In comparison, CHVs were engaged in part-time voluntary work, with greater schedule flexibility. Given their part-time work, they may have found it easier to incorporate TF-CBT delivery and supervision participation into their week. In another interesting comparison, CHVs’ estimates of how many hours TF-CBT required per week were nearly double those of teachers. Potentially, due to greater flexibility in CHVs tasks and voluntary work, it is possible for them to dedicate more time to preparation and delivery of the intervention. Alternately, given that most of the teachers had many years of teaching experience, their experience developing and delivering lesson plans may have allowed them to be efficient with their preparation time. It is notable that feasibility ratings were generally positive for lay counselors in both sectors. Findings from counselors in these first 10 schools and communities suggest that a multicomponent, multisession mental health intervention may be feasibly delivered by lay counselors in different sectors with varying levels of workload and competing demands.

Lay counselors in both settings generally reported that TF-CBT fit with them individually and their respective roles, as teachers or as CHVs. This high role appropriateness for two very distinct roles and sectors suggests that interventions such as TF-CBT may fit for lay counselors with varied task demands, primary roles, and time availability. Interestingly, there appears to have been more variability in lay counselors’ ratings of setting or context appropriateness of TF-CBT (compared to appropriateness for their own roles). All CHVs felt that TF-CBT would be useful for their community whereas teachers had positive, but more varied responses. Similarly, ratings of the fit of TF-CBT with one’s setting appear higher for CHVs, who are situated within the community health extension context, than for teachers, who are situated within the school context. This may reflect differences in feasibility (noted above) or may highlight the importance of considering the primary goals of a particular setting when moving mental health treatments into non-traditional settings. For instance, schools’ primary goals are academic, which may affect the implementation of interventions for which academics may not be the primary goal (47). Research increasingly supports the positive impact of mental health interventions on child academic outcomes (48, 49). Anecdotally, teachers who provided TF-CBT as part of BASIC shared stories of improved academic performance among children who received TF-CBT, and our team is seeking further funding to empirically examine academic outcomes for children who receive TF-CBT. With a new academic policy in Kenya encouraging a holistic education for children (50), mental health interventions may increasingly be perceived as a better fit for the Educational context.

Accurate and valid measurement is crucial to advance the fields of implementation science and global mental health, yet there are limited measures of implementation constructs and little research exists on their reliability or validity (28, 51–53). Valid and pragmatic measurement is essential to facilitating implementation and addressing stakeholder issues (54). We were able to use recently developed measures validated in the United States (29), measures specifically developed for task-shifted mental health interventions in LMICs (28), and the relatively recent Theoretical Domains Framework (42). While the Weiner measures were useful in assessing lay counselors’ perceptions of intervention acceptability and feasibility, they were not designed to provide insight on *why* lay counselors held these perceptions. The combined reflective and formative approach provided a way to both assess an underlying construct (e.g., 29) and obtain an understanding of what specific aspects led to it being perceived as acceptable, feasible, and appropriate. Use of similar approaches in other global mental health implementation studies may allow for some common measurement using reflective measures, but specificity from formative measurement. We felt the approach allowed for a deeper understanding of acceptability, feasibility and appropriateness in these two sectors, with some indication of implementation supports needed and how we might tailor support by setting. Findings from measures used in our approach mirror anecdotal evidence from counselors and coded qualitative interviews at post-implementation with a subset of these 60 counselors (55). Both teachers and CHVs have continued to deliver *Pamoja Tunaweza*/TF-CBT for a year following the post-implementation data collection point. As they have continued to deliver the intervention, they have made some adaptations to delivery to fit their context. In academic terms of shorter duration (term 3) counselors have delivered 2 groups in one week. Teachers and CHV counselors have developed strategies to support one another, based on their role and location, with teachers using their daily access to children (in the school/classroom) to remind guardians to attend groups by sending children home with reminder notes for their guardians. CHVs, who are embedded in the community, have been able to do more outreach with guardians who experience attendance problems (due to time constraints because of work or lower interest in group participation).

### Limitations

These findings should be considered within a number of study limitations. First, the findings are from only the first sequence in a larger SW-CRT. Our sample size in each sector is small. Results may be different after all 240 lay counselor participants are enrolled. However, the random selection and ordering process optimizes the validity of the selection of village clusters and the relatively consistent favorable scores across multiple measures of acceptability, feasibility, and appropriateness is encouraging. Second, due to the small sample, we did not consider it appropriate to conduct formal statistical tests of differences across lay counselors from the two sectors or to examine constructs that might predict low or high ratings of our constructs of interest. Third, internal consistency for the reflective measure of acceptability was just below the commonly held 0.70 thresholds and two IICs from the CHV surveys of feasibility were below the 0.20 threshold. These reflective measures hold promise given their brevity and seeming applicability, but more measurement work will be needed, with a larger sample, to determine if they are performing as expected with more diverse provider types and contexts. Fourth, while the JHU Implementation measures were designed for lay counselor delivery and use in low-resource contexts, some items required adaptation to fit our intervention and contexts and additional items were needed to capture aspects of feasibility and appropriateness considered important by the study team. The tension between using standardized measures when possible, and adapting and tailoring measures to fit the context is a difficult one to balance. Following recommendations of Martinez et al. (52), we have attempted to be clear about any measure adaptations in our measures section and included all items in our tables. We also plan to do more psychometric work on our measures with the full sample of lay counselors. Finally, findings from this study may not generalize to different regions or schools (e.g., secondary schools), given our desire to situate the study within the Kenyan context, and specifically, their community health extension program. Our lay counselor sample may also limit generalizability, as many of them indicated receiving some prior psychosocial training. Prior experience may have influenced lay counselors’ perspectives on the intervention’s acceptability, feasibility, and appropriateness.

## Conclusions

Task-shifting mental health care provides an approach for addressing the shortage of mental health professionals globally. In this study in western Kenya, lay counselors in both the Education and Health sectors found an evidence-based treatment for children and adolescents who had experienced parental death, TF-CBT, to be highly acceptable, feasible, and appropriate. Our findings suggest that, at least in terms of perceptions of the lay counselors delivering the intervention, despite differences in the lay counselors’ roles and context, both sectors hold promise as potential options for scaling up mental health treatment delivery.

## Data Availability Statement

The datasets for this manuscript are not publicly available because we cannot guarantee protection of study subjects’ confidentiality if we make the raw data publicly available. We are investigating ways to create limited de-identified datasets that maintain participant confidentiality for broader use upon completion of the study. Datasets are available upon request to the MPIs.

## Ethics Statement

The studies involving human participants were reviewed and approved by Duke University Institutional Review Board and the Kenya Medical Research Institute Institutional Review Board. Written informed consent to participate in this study was provided by the participants’ legal guardian/next of kin.

## Author Contributions

SD, RM, PM, NT, GW, CS, CA and CG wrote the paper. SD, KW, CS, and AW conceptualized the overall study. CG analyzed the data. SD, RM, PM, LL, NT, CG, CS, CA, AW, and KW interpreted the results and contributed to the construction of the *Discussion* section.

## Funding

Funding for this research project was supported by a grant to the first and last authors by the National Institute of Mental Health (R01 MH112633; Dorsey and Whetten, MPIs).

## Conflict of Interest

The first author, SD, and last author, KW, have received grant funding to test TF-CBT.

The remaining authors declare that the research was conducted in the absence of any commercial or financial relationships that could be construed as a potential conflict of interest.
